# Unequal Relief: Sex Disparities in Opioid Use for Cardiac Chest Pain in the Emergency Department

**DOI:** 10.5811/westjem.50599

**Published:** 2026-04-08

**Authors:** Jeffrey Druck, Dilan Al Kurdi, Mohamed Shubair, Radi Ahlat, Taryn Tenaya Hunt-Smith, Raed Darwish, Emad Awad

**Affiliations:** * University of Utah, School of Medicine, Department of Emergency Medicine, Salt Lake City, Utah; † University of Utah, School of Medicine, Salt Lake City, Utah; ‡ University of British Columbia, School of Medicine, Vancouver, British Columbia, Canada; § University of Utah, Department of Psychology, Salt Lake City, Utah; || Ain Shams University, School of Medicine, Cairo, Egypt; # University of British Columbia, Department of Emergency Medicine, British Columbia Resuscitation Research Collaborative, Vancouver, British Columbia

## Abstract

**Introduction:**

Acute chest pain, commonly caused by coronary artery disease, is a frequent reason for emergency department (ED) visits. While sex disparities in the evaluation and treatment of chest pain are well known, there is limited research on sex differences in the use of opioid analgesics for this condition in the ED. In this study we aimed to evaluate sex differences in the administration of opioid analgesics (morphine and fentanyl) and to compare the time to medication administration in patients presenting with acute cardiac chest pain.

**Methods:**

This retrospective observational study included adult patients (≥ 18 years of age) presenting with acute cardiac chest pain and confirmed elevated troponin between 2019–2024. The primary outcome was receipt of intravenous (IV) morphine and/or IV fentanyl. The secondary outcome was time from medication order to administration. For male vs female comparisons, we used t-tests or Mann-Whitney U tests for continuous variables, and chi-square tests for categorical variables. Logistic and linear regression analyses were performed to assess sex differences in opioid administration and time to medication, adjusting for potential confounders.

**Results:**

A total of 2,168 patients were included in the study, with 924 females (42.6%). Among morphine recipients, the median initial IV morphine dose was 5 mg (interquartile range [IQR] 4–5 mg; range 2–6 mg). Males had higher adjusted odds of receiving morphine compared to females (adjusted odds ratio [OR] 1.28, 95% CI, 1.04–1.57, P = .02). Females had a longer unadjusted time from order to morphine administration (median 11 minutes [IQR 6–20] vs 9 minutes [IQR 4–17]; P = .003). Time to fentanyl administration did not differ by sex. In adjusted analyses, there were no significant sex differences in time to morphine or fentanyl administration.

**Conclusion:**

This study identifies significant sex disparities in the administration of morphine to patients with acute chest pain. After adjusting for other factors, male patients had higher odds of receiving IV morphine compared to females. These findings highlight the need for further research to understand the underlying causes of these disparities and to develop strategies to ensure equitable chest pain management in the ED.

## INTRODUCTION

Acute chest pain is a common presenting symptom of coronary artery disease and myocardial infarction (MI), ranking among the top chief complaints in the emergency department (ED), with approximately half of the cases occurring in females.^1^,^2^ Numerous studies have documented sex disparities in the evaluation and treatment of chest pain in EDs.^2^–^4^ An analysis using data from the U.S. Centers for Disease Control and Prevention’s National Hospital Ambulatory Medical Care Survey (2014–2018) found that women presenting with chest pain experienced longer wait times and were less likely to receive electrocardiograms than men.^2^ Similarly, among over one million patients with acute coronary syndromes (ACS), women experienced longer delays from symptom onset to first medical contact and were less likely to receive statins, dual antiplatelet therapy, or percutaneous coronary intervention (PCI).^4^,^5^ Additional evidence has shown that women are less likely to undergo troponin testing^6^ or receive essential cardiac medications, including beta-blockers, lipid-lowering agents, and ACE inhibitors at discharge.^7^ Disparities in emergency care for women have also been reported in other cardiovascular emergency conditions such as cardiac arrest^8^,^9^ and atrial fibrillation.^10^

Previous studies have examined sex differences in the diagnostic and procedural management of chest pain in the ED, including delays in physician evaluation, cardiac enzyme testing, and receipt of invasive procedures such as PCI or discharge medications.^2^–^4^,^6^,^11^,^12^ While sex disparities in pain management have been documented more broadly, research specifically addressing disparities in opioid analgesia for cardiac chest pain in North American EDs remains limited.

Morphine and fentanyl are commonly used opioids for treating acute chest pain in ACS. Current guidelines recommend both as first-line options for patients with ongoing ischemic pain that is unresponsive to anti-ischemic therapy.^13^ Fentanyl is increasingly used as an alternative, particularly in patients with contraindications to morphine, such as hypotension or morphine intolerance.^13^–^15^ Our primary objective in this study was to evaluate sex differences in the administration of opioid analgesics, specifically morphine and fentanyl, in ED patients presenting with acute chest pain. The secondary objectives were to compare time to opioid administration and describe and compare key baseline and process measures, including triage acuity, door-to-doctor time, disposition, and nitroglycerin use by sex.

## METHODS

### Setting and Population

This was a retrospective observational study conducted in the ED of the University of Utah Hospital, a tertiary academic medical center in Salt Lake City, Utah, with approximately 62,000 annual visits. The study site uses the high-sensitivity troponin I (hs-TnI) assay, with elevated troponin defined as values > the 99th percentile upper reference limit (URL): > 34 nanograms per liter (ng/L) for men and > 16 ng/L for women, per manufacturer guidelines. Patients were included if they met the following criteria: 1) age ≥ 18 years; 2) triage documentation of “acute chest pain” as the chief complaint (reflecting patient-reported symptom onset within hours to days, without a fixed threshold); 3) a final ED discharge diagnosis consistent with ACS or MI (eg, ST-elevated myocardial infarction (STEMI), NSTEMI, unstable angina, MI) as reported in the ED discharge report or admission diagnosis; and 4) at least one elevated troponin level measured during the ED encounter. Exclusion criteria were as follows: patients with elevated troponin attributable to chronic kidney disease, sepsis, heart failure, and trauma-related chest pain; patients with documented allergies to morphine or fentanyl; and patients with missing data on sex or other key variables. For individuals with multiple visits for chest pain during the study period, only the first visit was included. We operationalized the ACS/MI status using the recorded final ED discharge diagnosis (and associated diagnosis terms/codes) at the time of ED disposition (ED discharge or hospital admission). We did not perform independent adjudication beyond the recorded ED diagnosis.

**Population Health Research Capsule**

What do we already know about this issue?

*Sex disparities exist in cardiac care, but evidence regarding opioid use for chest pain in the emergency department (ED) is limited*.

What was the research question?

*Do sex differences exist in opioid administration for cardiac chest pain in the ED?*

What was the major finding of the study?

*Males had higher adjusted odds of receiving morphine (aOR 1.28, P =.02) but not fentanyl*.

How does this improve population health?

*This study highlights a potential bias in analgesic choice, advocating for standardized protocols to ensure equitable pain relief for all chest pain patients*.

### Data Collection and Variable of Interest

Patient data were extracted from electronic health records (EHR). Variables included demographics (age, sex, race/ethnicity), vital signs, Emergency Severity Index (ESI) triage level, ED length of stay, door-to-doctor time, administration of opioid analgesics, and time from medication order to administration. The main independent variable was sex (male vs female). Sex was defined using the administrative sex field in the EHR, which is typically based on government-issued identification or insurance information. The primary outcome was the administration (yes/no) of intravenous (IV) morphine and IV fentanyl. The secondary outcome was the time (in minutes) from medication order to administration. This retrospective chart review adhered to key methodological standards recommended by Worster et al (2005),^16^ including defining clear inclusion and exclusion criteria, using a standardized abstraction form, training data abstractors, blinding abstractors to the study objective and hypothesis, monitoring performance, and identifying the medical record database. This study was deemed exempt from institutional review board (IRB) review by the University of Utah (IRB: 00094185) as it involved secondary analysis of de-identified data and posed minimal risk to patients.

### Statistical Analysis

We summarized baseline characteristics for the overall cohort and by sex. Continuous variables were presented as means with standard deviations (SD) or medians with interquartile ranges (IQR), depending on the distribution. Categorical variables were summarized as frequencies and percentages. We performed group comparisons using independent samples *t*-tests or Mann-Whitney U tests for continuous variables and chi-square tests for categorical variables.

For patients who received morphine, we extracted the initial administered IV morphine dose from the medication administration record and summarized it descriptively using the median (IQR) and range. To examine the association between sex and medication administration, logistic regression was used for binary outcomes (eg, whether opioids were administered). For continuous outcomes (eg, time to medication administration), we used linear regression. Prior to conducting multivariable regression, all assumptions, including multicollinearity among independent variables,^17^–^19^ were assessed. We used a forward selection approach to build adjusted models from candidate covariates, prioritizing clinically relevant variables and those associated with outcomes in unadjusted analyses. Model fit for both logistic and linear regression models was evaluated using the Akaike Information Criterion (AIC).^20^ The specific covariates included in each adjusted model are detailed in Tables 2–4. Because time-to-administration outcomes were right skewed, we conducted a sensitivity analysis using a generalized linear model with a gamma distribution and log link; exponentiated coefficients were interpreted as ratios of mean time.

As a sensitivity analysis, we repeated the regression analyses after excluding patients who were discharged directly from the ED and those who left against medical advice, as they were presumed to be clinically stable and less likely to require opioid analgesia. We also excluded patients who were acuity level 1 and/or died in the ED, as they were likely unconscious or critically ill and, therefore, unlikely to receive analgesia. All statistical tests were two-sided, with a significance level set at *P* < .05. Analyses were performed using SPSS Statistics v30 (IBM Corporation, Armonk, NY).

## RESULTS

### Baseline Characteristics, Process Measures, and Unadjusted Outcomes

We included 2,168 patients in the analysis: 1,244 males (57.4%) and 924 females (42.6%). Overall, 925 (42.7%) received IV morphine; among morphine recipients, the median initial IV dose was 5 mg (IQR 4–5 mg; range 2–6 mg). In line with our secondary objective to describe process measures, key differences by sex were observed (Table 1). Compared to males, females experienced longer door-to-doctor times (median 15.8 vs 13.7 minutes, *P* < .001), were less frequently triaged as high acuity (Level 1/2: 35.8% vs. 51.0%, *P* < .001), and were admitted less often (83.1% vs 85.9%, *P* < .01). Females were also less likely to receive sublingual nitroglycerin (37.6% vs 48.7%, *P* < .001) and had longer delays in receiving both nitroglycerin and morphine. No significant sex differences were observed in crude opioid administration rates (Table 1).

### Adjusted Results

#### Association Between Sex and Morphine Administration

The unadjusted comparison for the primary outcome showed no significant sex difference in morphine administration (42.0% vs 43.6%, *P* = .24; crude OR 0.94). However, the differences in baseline and process measures described above, particularly the marked disparity in triage acuity, represented significant potential confounders. To test the independent association of patient sex with opioid administration, we performed multivariable regression. After adjusting for these demographic and clinical variables, male sex was associated with significantly higher odds of receiving morphine (adjusted OR 1.28, 95% CI, 1.04–1.57, *P* = .02). In addition to male sex, higher triage acuity levels were significantly associated with morphine administration. The analysis also showed that receiving nitroglycerin prior to morphine was strongly associated with a reduced likelihood of morphine use (OR 0.08, 95% CI, 0.06–0.09, *P* < .001), indicating a 92% reduction in the odds of receiving morphine when nitroglycerin was administered first (Table 2).

#### Association between sex and fentanyl administration

The multivariable logistic regression analysis revealed that male sex was not significantly associated with receiving fentanyl in the crude model (crude OR 1.06, 95% CI, 0.81–1.39, *P* = .67). After adjusting for other variables, the association remained non-significant (adjusted OR 0.92, 95% CI, 0.69–1.22, *P* = .56) (Table 2).

#### Subgroup Analysis

In this subgroup analysis, we excluded 210 patients from the original sample of 2,168: 168 who were discharged directly from the ED; 10 who died in the ED (the majority of whom were triage level 1); and 32 who left against medical advice. These patients were excluded because they were either clinically stable (those discharged from the ED) or not suitable candidates for opioid analgesia (those who died in the ED). We included the remaining 1,958 patients (1,147 males and 811 females) in the analysis.

The results revealed a significant association between sex and the odds of receiving IV morphine. Specifically, male sex was associated with a significantly higher OR of 1.30 (95% CI, 1.05–1.62, *P* = .02). This indicates that male patients are 30% more likely to receive IV morphine compared to female patients after adjusting for other factors (Table 3). These findings are consistent with the primary analysis in both the direction and magnitude of the relationship between sex and receiving IV morphine. The subgroup analysis also showed that male sex was not significantly associated with the odds of receiving IV fentanyl (OR 1.10; 95% CI, 0.80–1.49; *P* = .56), indicating no substantial difference between male and female patients (Table 3). These findings are also consistent with the primary analysis results.

#### Time to Morphine

The multivariable linear regression analysis showed that, in the unadjusted model, males had a non-significantly shorter time to morphine administration (B = −2.16 minutes, 95% CI, −4.94–0.63, *P* = .13). This suggests that, on average, male patients received morphine 2.16 minutes sooner than female patients, although this difference was not statistically significant. After adjusting for covariates, the association between sex and morphine administration time remained non-significant (B = −2.56 minutes, 95% CI, −5.43–0.31, *P* = .08) (Table 4). In a sensitivity analysis using a generalized linear model with a gamma distribution and log link, results were consistent (female vs male ratio 1.14, 95% CI, 0.92–1.40, *P* = .24).

#### Time to Fentanyl

The analysis also demonstrated that males had a shorter time to fentanyl administration than females (unadjusted B = −2.62, 95% CI, −9.72–4.48, *P* = .47). This difference was not statistically significant. After adjusting for clinically relevant variables, the association remained non-significant (B = −1.91, *P* = .64), indicating no meaningful sex-based difference in time to fentanyl administration (Table 4). Gamma (log-link) sensitivity analyses yielded similar conclusions.

All assumptions for linear and logistic regression were tested and met. The models were then optimized using the AIC, with the final models having the lowest AIC values, indicating an improved fit compared to the other initial models.

## DISCUSSION

This study reveals a two-stage sex disparity in the emergency care of ACS. First, women were significantly less likely to be triaged as high acuity. Second, and critically, after adjusting for this and other clinical factors, male sex was independently associated with higher odds of receiving morphine. This suggests that bias may affect both the initial assessment of severity and the subsequent analgesic choice for patients at similar clinical urgency.

The subgroup analysis (excluding patients who were discharged, left against medical advice, or died in the ED) yielded results consistent with the primary analysis. While triage acuity differed by sex, adjusting for acuity showed that disparities in morphine use were not fully explained by clinical severity. Some residual confounding may remain. By focusing on a clinically homogeneous cohort, we minimized misclassification bias common in prior studies involving heterogeneous populations, 3,6,21 enhancing confidence in our findings. Nonetheless, further research is needed across diverse settings to confirm these results.

Importantly, fentanyl use did not differ significantly between sexes in either unadjusted or adjusted models. This suggests that women who did not receive morphine often received fentanyl instead, indicating the disparity may lie in the choice of opioid rather than overall analgesia. A randomized controlled trial from 2016 found no difference in pain scores or hypotension between groups receiving fentanyl and morphine.^22^ The 2025 American College of Cardiology/American Heart Association Joint Committee on Clinical Practice Guidelines recommends morphine or fentanyl as comparable agents in pain relief for acute coronary syndrome only after administration of other anti-ischemic medications.^13^ Thus, the fact that fentanyl use did not differ between sexes likely does not lie in differences in nationwide protocol, although it may reflect a more standardized application of fentanyl protocols at certain institutions or consistent clinicians’ preferences for fentanyl in specific emergency clinical scenarios, regardless of patient sex. It is also noteworthy that the overall use of fentanyl was lower than that of morphine. The overall lower use of fentanyl compared to morphine should be considered when interpreting this pattern.

These findings align with prior research showing sex-based disparities in treatment, including underuse of ACE inhibitors^7^ and statins 23 in women. Several factors may contribute to such disparities: implicit bias in clinical decision-making; diagnostic uncertainty due to atypical presentations in women^24^,^25^; and clinician perceptions about opioid risk or pain tolerance. Prior studies have shown that clinicians often underestimate women’s pain, recommending psychological rather than pharmacologic treatment.^26^ In related ACS chest-pain work, opioid-prescribing patterns were not meaningfully different after adjustment and appeared to be driven more by clinical/process factors than clinician characteristics.^27^ It is worth noting that our analysis revealed a significant association between prior nitroglycerin use and reduced morphine administration, likely reflecting protocols that prioritize nitroglycerin for ischemic chest pain before escalating to opioids.^13^ Our analysis also showed that there were no significant sex differences in the time to morphine or fentanyl administration. While males received morphine and fentanyl slightly earlier than females, these differences were not statistically significant, even after adjusting for relevant variables.

The recommendation of this study includes incorporating educational sessions for healthcare clinicians to raise awareness about sex and gender bias in pain management. These sessions should focus on recognizing implicit bias, standardizing pain assessment protocols, and promoting equitable care to ensure all patients receive it. Educational sessions should stress equitable pain management despite diagnostic uncertainty. While distinguishing anxiety from ACS is important, clinicians must prioritize treating pain based on clinical presentation rather than waiting for confirmatory tests like troponin, which can delay analgesia, especially in women with atypical symptoms. Finally, consistent with other studies,^28^,^29^ Asian and Native American patients showed a trend toward lower rates of morphine administration, although small sample sizes limit definitive conclusions.

## LIMITATIONS

This study has several limitations. As a single-center, retrospective analysis, the findings may not be generalizable to other settings. Reliance on EHR data limits the ability to capture nuanced clinical decision-making or physicians’ rationale for medication use. Additionally, ACS/MI status was based on the recorded final ED discharge diagnosis rather than prospective adjudication; thus, misclassification was possible. Differential misclassification may occur if atypical presentations (more common among women) are less likely to be labeled ACS/MI, potentially influencing cohort inclusion and treatment patterns. Neither were we able to account for patient-reported pain scores, which may have influenced analgesic use. We lacked direct measures of ED crowding (eg, ED census/occupancy) and shift-level workload; therefore, residual confounding related to time-of-day/day-of-week variation and ED busyness may remain.

Further, we were unable to account for clinician-level variables such as years of experience. Additionally, although we adjusted for multiple confounders, residual confounding may still be present. Finally, our study did not assess the impact of morphine administration on patient-centered outcomes such as infarct size or survival, which prior research has shown to have minimal or no benefit in ACS.^30^–^33^

## CONCLUSION

In this retrospective cohort of patients presenting with acute chest pain and confirmed ACS, male patients had significantly greater adjusted odds of receiving IV morphine compared to females. However, no significant sex differences were observed in fentanyl administration or in the time to receive either morphine or fentanyl. Although unadjusted analyses suggested a small (~ two minutes) difference in time to morphine administration, adjusted models (including gamma sensitivity analyses) showed no significant sex difference; therefore, any clinical significance is uncertain, and we did not assess downstream patient-centered outcomes. These findings suggest the need for increased awareness of potential sex and gender bias in pain management in the ED and advocate for further research to standardize analgesia protocols, ensuring equitable care for all patients.

## Figures and Tables

**Table 1:** Demographics in a retrospective study of adults presenting to the emergency department with cardiac chest pain in a study of sex disparities in the administration of morphine to patients with acute chest pain.

Variable	Total (N = 2,168)	Male (n = 1,244)	Female (n = 924)	P-value
Age (mean [SD])	60.4 (11.7)	60.9 (14.5)	59.8 (16.1)	.11
Race/Ethnicity (N, %)				
White	1,602 (73.9)	936 (75.2)	666 (72.1)	<.01
Hispanic or Latino	254 (11.7)	119 (9.6)	135 (14.6)	
Black	100 (4.6)	56 (4.5)	44 (4.8)	
Native American	83 (3.8)	47 (3.8)	36 (3.9)	
Asian	50 (2.3)	31 (2.5)	19 (2.1)	
Unreported/refused to report	79 (3.6)	55 (4.4)	24 (2.6)	
BMI (mean [SD])	30.5 (8.1)	30.1 (7.2)	31.0 (9.1)	.05
SBP (mean [SD])	143.7 (27.5)	143.8 (27.6)	143.6 (27.5)	.82
HR (mean [SD])	86.7 (21.0)	86.0 (21.2)	87.7 (20.8)	.08
RR (mean [SD])	18.7 (3.9)	18.9 (3.9)	18.6 (3.7_	.06
Door to doctor (median, IQR)	14.7 (7.5–32.7)	13.7 (7.0–28.0)	15.8 (9.0–38.0)	<. 001
Acuity level (N, %)				
Level 1	36 (1.7)	30 (2.4)	6 (0.6)	< .001
Level 2	929 (42.9)	604 (48.6)	325 (35.2)	
Level 3	1,203 (55.2)	610 (49.0)	593 (64.2)	
Type of initial treating clinician (N, %)				
Advanced practice practitioner	146 (6.7)	74 (5.9)	72 (7.8)	.09
MD or DO	2,022 (93.3)	1,170 (94.1)	852 (92.2)	
ED LOS/hours (median, IQR)	6.0 (4.25–8.0)	5.5 (3.8–7.5)	6.6 (4.8–8.7)	< .001
ED disposition (n, %)				
Admit	1,837 (84.7)	1,069 (85.9)	768 (83.1)	<.01
Against medical advice	32 (1.5)	16 (1.3)	16 (1.7)	
Discharge	168 (7.7)	74 (5.9)	94 (10.2)	
Expired	10 (0.5)	–	–	
Sent to operating room	59 (2.2)	39 (3.1)	20 (2.2)	
Transferred to another facility	62 (2.9)	39 (3.1)	23 (2.5)	
Medication administered (N, %)				
SL nitroglycerin: Yes	953 (44.0)	606 (48.7)	347 (37.6)	< .001
IV morphine: Yes	925 (42.7)	522 (42.0)	403 (43.6)	.23
IV fentanyl: Yes	242 (11.2)	142 (11.4)	100 (10.8)	.40
Time to first dose				
Nitroglycerin (median, IQR)	8.0 (4.0–16.0)	8.0 (4.0–14.5)	10.0 (4.0–20.0)	.02
Morphine (median, IQR)	10.0 (5.0–19.0)	9.0 (4.0–17.0)	11.0 (6.0–20.0)	<.01
Fentanyl (median, IQR)	8.0 (4.0–16.0)	7.0 (2.0–16.5)	9.5 (7.0–16.7)	.22

*SD*, standard deviation; *BMI*, body mass index; *SBP*, systolic blood pressure; *HR*, heart rate; *RR*, respiratory rate; *IQR*, interquartile range; *LOS*, length of stay; *MD*, Doctor of Medicine; *DO*, Doctor of Osteopathic Medicine; *SL*, sublingual; *IV*, intravenous.

**Table 2:** Crude and adjusted associations between sex and opioid administration in a study of sex disparities in the administration of morphine to patients with acute chest pain in the emergency department.

Variable	Morphine Administration OR (95% CI)	P-value	Fentanyl Administration OR (95% CI)	P-value
Male sex (crude)	0.94 (0.79–1.11)	.24	1.06 (0.81–1.39)	.67
Male sex (adjusted)	1.28 (1.04–1.57)	.02	0.92 (0.69–1.22)	.56
Age	1.00 (0.99–1.01)	.98	1.01 (0.99–1.02)	.24
Race (Ref: White)				
Native American	0.55 (0.32–0.96)	.03	1.30 (0.67–2.56)	.44
Black	1.33 (0.83–2.15)	.24	0.96 (0.49–1.86)	.90
Hispanic or Latino	1.04 (0.76–1.43)	.81	0.87 (0.55–1.38)	.56
Asian	0.47 (0.24–0.93)	.03	3.06 (1.5–6.01)	<.001
Unreported/Unknown	1.51 (0.88–2.62)	.14	0.78 (0.35–1.76)	.55
BMI	0.99 (0.97–1.01)	.09	1.01 (0.98–1.02)	.58
Acuity (Ref: Level 1)				
Level 2	4.02 (1.55–10.45)	.004	0.38 (0.08–1.81)	.22
Level 3	5.20 (2.00–13.52)	<.001	0.22 (0.05–1.08)	.06
Door-to-doctor time	0.99 (0.99–1.01)	.12	1.00 (1.00–1.01)	.73
MD/DO vs APP	1.01 (0.67–1.52)	.97	1.71 (0.85–3.45)	.13
SBP	1.01 (0.99–1.01)	.51	0.99 (0.98–0.99)	<.001
HR	0.99 (0.99–1.00)	.05	1.01 (1.00–1.02)	<.001
RR	0.99 (0.97–1.02)	.73	1.02 (0.98–1.05)	.29
Nitroglycerin prior to opioid	0.08 (0.06–0.09)	<.001	0.03 (0.02–0.07)	< .001
Other IV opioids	0.04 (0.02–0.05)	<.001	0.04 (0.02–0.06)	< .001

*OR*, odds ratio; *Ref*, reference category; *BMI*, body mass index; *SBP*, systolic blood pressure; *HR*, heart rate; *RR*, respiratory rate; *APP*, advanced practice practitioner; *MD*, Doctor of Medicine; *DO*, Doctor of Osteopathic Medicine; *IV*, intravenous.

**Table 3:** Crude and adjusted associations between sex and opioid administration in subgroup analyses (n = 1,958) in a study of sex disparities in the administration of morphine to patients with acute chest pain.

Variable	Morphine Administration OR (95% CI)	P-value	Fentanyl Administration OR (95% CI)	P-value
Male sex	1.30 (1.05–1.62)	.02	1.10 (0.80–1.49)	.56
Age	1.01 (0.99–1.01)	.63	1.01 (1.00–1.02)	<.01
Race (Ref: White)				
Native American	0.58 (0.33–1.04)	.06	1.51 (0.72–3.17)	.27
Black	1.22 (0.73–2.02)	.45	1.07 (0.53–2.14)	.85
Hispanic or Latino	1.10 (0.79–1.54)	.57	0.86 (0.51–1.44)	.56
Asian	0.58 (0.28–1.17)	.13	3.60 (1.68–7.70)	< .001
Unreported/Unknown	1.48 (0.84–2.58)	.17	0.72 (0.31–1.64)	.43
BMI	0.99 (0.98–1.01)	.07	1.03 (0.99–1.04)	.61
Acuity (Ref: Level 2)				
Level 3	1.41 (1.05–1.91)	.03	1.88 (0.6–4.10)	.23
Door-to-doctor time	1.01 (0.99–1.02)	.20	1.0 (0.98–1.02)	.56
MD/DO vs APP	0.84 (0.51–1.39)	.51	1.35 (0.5–3.08)	.47
SBP	1.01 (0.99–1.01)	.497	0.99 (0.99–1.00)	.11
HR	0.99 (0.99–1.01)	.062	1.01 (1.00–1.01)	.09
RR	1.01 (0.97–1.03)	.939	0.99 (0.96–1.03)	.68
Nitroglycerin prior to opioid	0.08 (0.06–0.11)	<.001	0.12 (0.08–0.19)	<. 001
Other IV opioids	0.03 (0.02–0.06)	<.001	0.04 (0.01–0.05)	< .001

*OR*, odds ratio; *Ref*, reference category; *BMI*, body mass index; *SBP*, systolic blood pressure; *HR*, heart rate; *RR*, respiratory rate; *APP*, advanced practice practitioner; *MD*, Doctor of Medicine; *DO*, Doctor of Osteopathic Medicine; *IV*, intravenous.

**Table 4:** Crude and adjusted association between sex and time to opioids administration in a study of sex disparities in the administration of morphine to patients with acute chest pain.

Variable	Time to Morphine		Time to Fentanyl	
	
	B Coef (95% CI)	P-value	B Coef (95% CI)	P-value
Male sex (unadjusted)	−2.16 (−4.94, −0.63)	.13	−2.62 (−9.72, 4.48)	.47
Male sex (adjusted)	−2.56 (−5.43, −0.31)	.08	−1.91 (−9.96, 6.15)	.64
Age (per year)	0.07 (−0.02; −0.16)	.15	−0.08 (−0.32, 0.16)	.50
Race (Ref: White)				
Native American	−0.94 (−9.53, −7.66)	.83	−4.00 (−20.90, 12.93)	.68
Black	1.08 (−5.37, −7.52)	.74	−9.79 (−24.34, 6.75)	.24
Hispanic or Latino	−3.32 (−7.72, −1.07)	.14	5.30 (−7.2, 16.83)	.40
Asian	−1.04 (−11.02, −9.93)	.84	−0.70 (−15.38, 13.98)	.93
Unreported/Unknown	0.59 (−6.17, −7.34)	.87	−9.40 (−24.31, 9.58)	.33
BMI	−0.08 (−0.29, −0.12)	.43	0.07 (−0.28, 0.41)	.71
Door-to-doctor time	0.04 (0.006, −0.06)	.02	0.04 (−0.08 – 0.16)	.54
MD/DO vs APP	−3.04 (−8.73, −2.66)	.29	−2.84 (−18.67, 12.99)	.72
SBP	0.11 (−0.02, −0.25)	.10	0.07 (−0.05, 0.19)	.25
HR	0.04 (−0.13,−0.21)	.64	0.05 (−0.11, 0.20)	.55
RR	−0.67 (−1.52, −0.19)	.13	−0.82 (−1.61,−0.04)	.04
Nitroglycerin prior to opioids	−3.63 (−9.25, −1.99)	.21	−8.17 (−24.19. 7.85)	.31

*B Coef*, regression coefficient; *Ref*, reference category; *BMI*, body mass index; *SBP*, systolic blood pressure; *HR*, heart rate; *RR*, respiratory rate; *APP*, advanced practice practitioner; *MD*, Doctor of Medicine; *DO*, Doctor of Osteopathic Medicine.
